# Zones of (un)certainty: studying the temporal stabilization of ground truths for medical AI

**DOI:** 10.1007/s00146-026-03228-x

**Published:** 2026-08-07

**Authors:** Dieuwertje Luitse, Tobias Blanke

**Affiliations:** University of Amsterdam, Amsterdam, The Netherlands

**Keywords:** Zones of (un)certainty, Ground truth, Ground-truth studies, Data annotation, Medical AI

## Abstract

Medical data annotation is a critical yet contingent process that (re)configures ambiguous medical decisions and data representations into ground truths for artificial intelligence (AI). Extending critical data, AI, and STS research on uncertainty in data annotation, this article develops the concept of *zones of (un)certainty* to capture the epistemic time-spaces through which annotators transform uncertainty and dissonance into stabilized ground-truth data for medical AI. Drawing on interviews with AI and medical researchers at two Dutch University Medical Centres and complemented by observations at an interdisciplinary workshop on annotation practices, we empirically unfold three such *zones*: first, we identify an epistemic time–space where ground-truthing is approached spatially as practitioners decompose and recompose data layers, views, and metadata to reach “good enough” certainty. Second, we analyze temporal zones of drift where micro-judgments are routed through negotiations with colleagues, experts, AI suggestions, and automated evaluations. Third, we examine thresholding practices in which uncertain cases are gradually compressed into singular labels. Taken together, this article provides insights into the temporal–spatial dimensions and thresholds that annotators use to settle on ground-truth decisions. Consequently, we open a broader analytical vocabulary for studying how uncertainty and dissonance are operationalized for AI-based medicine and beyond.

## Introduction

It is a Monday morning in March, and we are looking at a computer screen displaying cardiac computed tomography (CT) scans to detect and segment arterial calcium structures. “So, in one case,” the annotator I am speaking to explains, “it might look like the calcium is in this organ. But in [an]other scan, it might not be there because the patient is in a different position.” They scroll through the image layers and continue: “Each [image] slice is 3 millimeters thick. If we thus move 3 millimeters to the left or the right, the appearance of calcium [suddenly] changes.”

As the conversation illustrates, the manual annotation of medical data (e.g., computed tomography (CT), electrocardiograms (ECG), or electronic health records) for the development of artificial intelligence (AI) is a critical yet contingent process, (re)configuring ambiguous medical decisions and bodily representations in multimodal medical data into ground truths for AI (Jaton 2017). Ground truths encompass the relational, referential datasets that gather “input-data and [human-labeled] output-targets” (Jaton 2023, p. 788), which contain the information the AI system is intended to process as well as the results the system is expected to produce (Kang and Hudson 2024; Rettberg 2024). Such ground-truth datasets set the conditions for “a machine learning system to build its own model based on the parameters, patterns, associations, and weights that are derived from processing the ground truth” (Sadowski 2026, p. 5). The model is then evaluated against a similar type of ground truth to optimize the system further and improve its performance (Luitse et al. 2025). Ground-truth datasets are considered key infrastructure (Thylstrup 2022), forming the underlying conditions for knowledge production on which (supervised) AI systems operate (Denton et al. 2021).

In contrast to ground-truth construction in other fields in computer vision, where the labor is often outsourced via micro-working platforms (Miceli et al. 2020) or conducted by computer scientists in labs (Engdahl 2024), annotations for the development of medical AI systems and applications are generally provided by teams of medical experts like radiologists or cardiologists, medical or medical AI PhD candidates or other medical students. Such domain- or expert-labeled ground truth can be challenging to obtain due to the time and labor involved. Processes of defining and applying specific data labels are complex and unstable e.g., due to the multi-dimensionality of the data as in the opening quote and because AI and medical decision-making practices are particularly ambiguous and uncertain (e.g., Timmermans and Angell 2001; Bennett 2025; Carboni et al. 2025). Additionally, medical annotators have diverse medical (or AI) backgrounds, areas of expertise, ways of conducting annotations and contexts in which they complete annotation tasks. This increasingly raises concerns about data reliability and the potential for harmful model biases (Luitse et al. 2025).

To address such issues, a growing body of critical data, AI, and science and technology studies (STS) scholarship investigates how ground truths are constructed across domains (e.g., Bennett 2025; Catanzariti 2025; Ratner and Thylstrup 2025), including medicine (Jaton 2023; Högberg 2025). This work draws on longstanding debates about how “truth” (Latour 1987) and “objectivity” (Daston and Galison 2007) are constituted and negotiated through epistemic uncertainties and dissonances intrinsic to scientific work (Star 1985; Forsythe 2001). In medical AI, such tensions emerge, for instance, from a “lack of base knowledge” (Bennett 2025, p. 2) and from incommensurable ways of knowing medical data subjects as practitioners interpret data through situated judgments. This resonates with the notion that medical bodies are enacted as “multiples” across practices and contexts (Mol 2002).

This existing research has highlighted how socio-technical conditions shape epistemic uncertainty in annotations and exposes the limits of ground truth as a stable basis for AI training and evaluation. However, limited work has subsequently explored how annotation work’s epistemic uncertainties and dissonances are temporally stabilized to become “certain enough” for ground-truth decisions. While Schjøtt and Blanke (2026) treat model development as settled once it is “good enough” based on diverse values, we instead concentrate on establishing when knowledge relations within the dataset are “certain enough”. The necessity to create ground truth compels annotators to analyze, negotiate, and transform emergent medical uncertainties into stabilized decisions that lead to a specific annotation. This paper addresses this gap by examining various ways in which annotating practitioners in medical AI research or particular medical specializations iteratively navigate different types of complex uncertainties and temporally materialize ground truths required for medical AI system development. We argue that, because these knowledge practices are relational and constantly evolving, we need a conceptual framework that captures the temporally structured epistemic environments, where annotators move from uncertainty and dissonance to settled ground-truth decisions.

To this end, we introduce and develop the concept of *zones of (un)certainty*. This concept foregrounds the hybrid, temporal, and interconnected knowledge spaces in which epistemic uncertainties and dissonances are continuously worked through and transformed in medical annotation practices to stabilize and materialize ground-truth decisions. As delineated, processual spaces for knowledge production, zones mark sites where heterogeneous epistemic practices overlap^1^ and ground-truth decisions are negotiated, as well as moments when ambiguous medical phenomena are rendered temporarily commensurable by individuals or teams of annotators. In this sense, zones function as epistemic time-spaces of interaction and transformation, where uncertainties about medical bodies are continuously gathered, reorganized, and reshaped to produce ground truth for medical AI. Approaching such practices of stabilization and materialization by conceptualizing zones of (un)certainty enables us to analyze the conditions under which certainty is reached.

Subsequently, we mobilize the concept of *zones of (un)certainty* through interviews with AI researchers and medical practitioners (MDs) involved in data annotation at two Dutch University Medical Centres, alongside observations from an international interdisciplinary workshop on data annotation practices at Medical Imaging with Deep Learning (MIDL). This empirical analysis demonstrates how ground truth(s) for medical AI are constructed and stabilized through and out of multiple, interconnected *zones of (un)certainty*.

In what follows, we first situate our contribution within the literature on uncertainty in ground‑truth construction for (medical) AI development. Second, we develop the notion of *zones of (un)certainty* by drawing on and extending Peter Galison’s (1997) concept of trading zones and Andrew Barry’s (2006) notion of technological zones. Trading zones describe socio-technical spaces in which heterogeneous epistemic communities develop local coordination that enables collaboration despite at least partially incommensurable languages, practices, and forms of knowledge. Technological zones, by contrast, are spaces in which coordination is achieved through infrastructures, standards, and technical protocols that align practices across sites and actors. While each concept highlights how heterogeneous scientific practices and knowledge systems are aligned and standardized, bringing them into conversation allows us to analyze how epistemic uncertainties in medical annotations are negotiated and transformed within specific epistemic time-spaces to construct meaningful ground truths. Our concept of *zones of (un)certainty* builds on these works. Still, it shifts the focus toward situated practices of working through competing interpretations of medical data and of organizing and temporarily stabilizing uncertainty and dissonance to produce ground‑truth labels considered “good enough” for AI development and evaluation. Finally, after outlining our methodological approach, we analyze practitioners’ annotation practices and identify three zones of (un)certainty that continuously reconfigure labeling decisions and shape ground‑truth construction in medical AI.

## Ground truth(s) and uncertainty

Contrary to popular claims to objectivity, scientific knowledge production is profoundly shaped by its situated experimental practice and through co-constitutive socio-technical processes (Latour and Woolgar 1986; Galison 1987; Galison and Buchwald 1995). This is no different for AI research and development and knowledge production, which is inherently experimental, iterative (Schjøtt and Blanke 2026), and configured by “ground truth data assemblages” (Högberg 2025, p. 85) for model training and evaluation. Originally understood as “remotely sensed data with real features and materials on the ground” (Mayhew 2015, p. 230), ground truth refers to the scientific search for “what is real, given organizational demands to produce useful information” (Fine 2006, p. 7). However, as socio-technical assemblages of data, human annotations (e.g., a particular medical diagnosis), and their underlying computational infrastructures, ground-truth for AI is not a stable referential basis. Its contingency shapes “how [domain] knowledge is constructed, contested, and codified” (Thylstrup and Hartley 2025, p. 106) through individual interpretations and decision-making points.

In an ongoing attempt to trace how subjective ground truths are constituted and operationalized in practice across various AI (sub-)disciplines - including medicine -, STS and critical data and AI scholars have developed critical inquiries, also called “ground truth studies” (Jaton 2025). Kang (2023, p. 2) has also referred to this qualitative research as “ground truth tracings” which allow us to think through the “sociotechnical assemblage of humans, machines, imaginaries, and infrastructures that ‘learnability’ [of a machine-learning model] is contingent upon”. More specifically, such interrogations provide empirical insights into the concrete and mundane knowledge practices underpinning ground-truthing processes, which include improvisational, arbitrary behavior and decision-making, ongoing negotiations of expertise and alignment work toward previously set standards (Jaton 2025; Lee 2025).

Catanzariti (2025), for example, shows how, when labeling facial expression recognition data, annotators intuitively interpret affective signals to ensure they fit predefined annotation schemes. In doing so, they continuously negotiate uncertain interpretations to achieve consistency and align with a shared truth framework required for model production. These negotiations and resulting disagreements are ultimately recast as technical glitches or statistical outliers under the “forces of scalability” that structure annotation work (Catanzariti 2025, p. 40). For medical AI annotation, Högberg (2025) shows that constructing a “workable” ground truth requires a pragmatic approach shaped by individual assumptions despite potential errors or incomplete data. The valuation of expert judgments is therefore marked by epistemic uncertainty as AI developers continuously assess the accuracy and trustworthiness of labeled data. This is particularly challenging because annotation reliability varies across hospitals and expert groups, while developers often lack the domain expertise to evaluate and compare annotation quality.

Together, these studies provide valuable empirical insights into how ground truth is contingent on annotation uncertainty and dissonance, which are inherent to situated (medical) AI development practices that strive to construct objectivity (Catanzariti 2025) and truth (Högberg 2025). However, the existing work pays limited attention to how annotators actively work through epistemic dissonances to render decisions sufficiently stable to count as ground truth. This article focuses on the temporal stabilization of such uncertainties to create “certain enough” data. We analyze the dynamic epistemic time-spaces that we call *zones of (un)certainty* through which annotators negotiate and transform uncertainty and dissonance into stabilized decisions. Before we present our empirical analysis, we first conceptualize *zones of (un)certainty*.

## Conceptualizing zones of (un)certainty

Z*ones of (un)certainty* are our conceptual framework for empirically capturing the socio-technical epistemic time-spaces that emerge as data annotators practically transform uncertain and differing (micro-)judgments into temporally stabilized ground-truths. For this framework and accompanying empirical analysis, we draw on, adapt, and extend two influential STS conceptualizations of zones: (1) Trading Zones (Galison 1997); and (2) Technological Zones (Barry 2006).

“A trading zone”, Galison (1997, p. 781) explains, “is a ‘hybrid’ site where different actors collaborate, although they do not fully understand each other and do not interpret the world in the same manner.” This idea has proved valuable for conceptualizing the challenges and opportunities that arise when diverse sets of scientific expertise come together (e.g., AI research and medicine). “Central to the idea of the trading zone”, Hine and Barnaghi (2024, p. 4) explain, “is that different disciplines may have incommensurable ways of understanding a problem—different languages in effect—and that for them to collaborate on a project, some locus of cooperation or trading zone has to develop.” Within such spaces of negotiation, disparate groups can collaborate and develop shared but localized forms of communication or a shared language to interact and understand each other’s research values, assumptions, and concerns. As medical data annotations require different actors and expertise to come together around the same problem, annotating actors operate similarly. AI researchers and medical practitioners, e.g., operate according to different values and practices (see Luitse (2026), for an extensive analysis on this topic). Yet, to produce the necessary ground truths for AI, they nonetheless create epistemic environments—the trading zones—to work together “in the absence of a full-blown translation” (Galison 1997, p. 833). Medical AI thus unfolds within what Cambrosio et al. (2006) call a regime of “regulatory objectivity”, where evidence is generated through concerted programs of collective action that produce entities, protocols, and standards for judgment. Rather than seeking a single “true value”, regulatory objectivity aims to ensure the compatibility and comparability of results across laboratories, hospitals, and platforms by stabilizing conventions to make medical classification “certain enough”.

In addition to trading zones, medical data annotators arguably operationalize technological zones to stabilize ground truths for AI development and evaluation. “A technological zone”, Barry (2006, p. 239) describes, “can be understood as a space within which differences between technical practices, procedures and forms have been reduced, or common standards have been established”. Such technological zones take broadly one of three forms: (1) metrological zones associated with the development of common forms of measurement; (2) infrastructural zones associated with the creation of common connection standards; and (3) zones of qualification which come into being when objects and practices are assessed according to common standards and criteria (Barry 2006). In determining a final ground-truth label, medical data annotators arguably operate within such technological zones, as they align their practices with medical standards developed through theory, individually or collectively negotiate annotation protocols and validation criteria, and use the same annotation tools. By doing so, this type of data annotation work involves the ongoing reduction of individual and collective dissonances in medical interpretations using, e.g., guidelines, collective agreements, and quality assessment procedures that render heterogeneous judgments between annotators or medical experts negotiable and comparable ground-truth production.

The concepts of trading zones and technological zones thus provide distinct insights into how scientific practices from different disciplines and situated ways of knowing are aligned and standardized to produce large volumes of medical data. However, as annotation work for medical AI is both contingent on collaboration among actors from various scientific disciplines and on reducing medical and technical differences in the service of ground-truth production, we have to integrate and further extend these insights into our framework of zones of (un)certainty. In our work, these zones should not simply be understood as the locations where the technical work of data labeling for medical AI takes shape. Each zone we identify represents a socio‑technical epistemic time‑space in which different forms of certainty, uncertainty, and dissonance are negotiated and temporarily stabilized within medical annotation practices. Within these zones, ground‑truth data are constructed and become “good enough” for training and evaluating AI systems for medical applications. Through careful analytical observation of these zones of (un)certainty, we contribute to STS scholarship on how certainty, uncertainty, and dissonance materialize in ground‑truth production. As such, the framework enables us to highlight the temporal knowledge spaces that different types of medical annotators construct when abstract ideas in ground‑truth data must be operationalized and rendered actionable for model training and evaluation.

## Data and methodology

To understand the emergence of specific *zones of (un)certainty* in the production of ground-truth data for medical AI research and development, this paper draws on two unique sets of data: the first one is a set of transcripts of 12 in-depth interviews with AI researchers in medicine (PhD level) and junior medical practitioners (MDs) who are conducting data annotations for the production of ground-truth datasets for AI research in Radiology, Cardiology, and Intensive Care at two Dutch academic medical centers. Our interviewees worked either individually or in a team setting on a variety of annotation projects (see Table 1 for a full overview). As we were a part of a research project on Responsible AI for Health Decision-making, as well as a national project to create responsible AI applications, all interview participants were recruited through our professional networks and snowball sampling. The interviews were conducted between March and June 2025, took place in either English or Dutch, and lasted between 40 and 60 min. All materials were pseudonymized and transcribed using the transcription software Amberscript, then manually checked and edited for clarity and readability. The respective medical data types included imaging data (computed tomography (CT); computed tomography angiographies (CTA); and optical coherence tomography (OCT)); electronic health records (Tabular Data) and signals (ECG data). Our second dataset comprises results and observations from an interdisciplinary workshop on data annotation practices organized by the first author together with a medical AI colleague in collaboration with Medical Imaging with Deep Learning (MIDL). MIDL is an international research community and conference that brings together medical imaging and AI researchers, clinicians, and healthcare companies to address specific research problems and explore new direction (Medical Imaging with Deep Learning 2026).

**Table 1:** Overview of participants, including project and department affiliations

ID	Role	Project	Department
001	PhD Candidate Intensive Care Medicine (MD)	Annotation of blood pressure data (Electronic Health Records) for the prediction of complications with ICU patients after major surgery	Intensive Care Medicine
002	PhD Candidate AI Research for Medical Imaging	Segmentation mask annotation of coronary artery calcium (CAC) lesions in cardiac CTs	Biomedical Engineering and Physics; Institute of Informatics
003	PhD Candidate AI Research for Medical Imaging	Segmentation mask annotation of bone structures and knee implants in knee CTs Segmentation mask annotation of bone structures in wrist and shoulder CTs	Biomedical Engineering and Physics; Institute of Informatics
004	PhD Candidate AI Research for Medical Imaging	Annotation of artery bifurcation points in Runn-off CTAs	Biomedical Engineering and Physics; Institute of Informatics
005	PhD Candidate Intensive Care Medicine (MD)	Annotation of Atrial Fibrillation in 12-lead ECGs	Intensive Care Medicine
006	PhD Candidate Intensive Care Medicine (MD)	Annotation of Electronic Health Records for a Dutch National ICU database	Intensive Care Medicine
007	PhD Candidate AI Research for Medical Imaging	Segmentation mask annotation of intravascular structures and lesions in OCT images at a cardiac catheterization lab	Cardiology
008	PhD Candidate AI Research for Medical Imaging	Segmentation mask annotation of intravascular structures and lesions in OCT images at a cardiac catheterization lab	Cardiology
009	PhD Candidate AI Research for Medical Imaging	Segmentation mask annotation of intravascular structures and lesions in OCT images at a cardiac catheterization lab	Cardiology
010	PhD Candidate Intensive Care Medicine (MD)	Annotation of Electronic Health Records for a Dutch National ICU database	Intensive Care Medicine
011	PhD Candidate AI Research for Medical Imaging	Annotation of coronary intravascular stents in OCT images	Cardiology; Biomedical Engineering and Physics; Institute of Informatics
012	PhD Candidate Intensive Care Medicine (MD)	Annotation of Electronic Health Records for a Dutch National ICU database	Intensive Care Medicine

In conducting the interviews, we integrated think-aloud elements (Eccles and Arsal 2017). The participants provided us with visual examples of the annotation process that we could observe and “thought aloud” while producing an annotation task. This means participants would, e.g., describe what they looked at in a medical image and how they eventually decided on a particular data label (e.g., a segmentation mask). This interview technique, substantiated by follow-up conversations about uncertainties and dissonances in the creation of data labels (e.g., between individuals themselves, between team members, or between annotators and other types of medical experts), provided us with insights into the micro-judgments and micro-decisions that shape the materialization of medical annotations within the various projects. Building upon these insights, it also allowed us to distill the specific socio-technical environments through which epistemic uncertainties and dissonances are constitutively implicated in medical annotation practices and in the (temporal) stabilization of ground-truth data for medical AI.

We analyzed the data using a reflexive thematic analysis approach (Braun and Clarke 2019, 2021). This widely adopted variant of thematic analysis (Braun and Clarke 2019) enabled the identification, analysis, and reporting of themes or patterned meaning within data (Braun and Clarke 2021). At the same time, it allowed us to remain flexible enough to engage with emergent themes and situate our findings within wider socio-cultural contexts of knowledge production for medical AI development and application. The analysis was conducted through an iterative, inductive, and generative process using the qualitative coding software Atlas.ti alongside a close reading of printed interview transcripts. Braun and Clarke’s approach allowed us to identify, analyze, and report on the different *zones of (un)certainty* in data annotation practices that we could not have foreseen in advance. The research was approved by the research ethics committee of the Faculty of Humanities at the University of Amsterdam (FGW-4538).

## Emerging zones of (un)certainty in medical data annotation

Across the medical AI projects considered in this research, creating annotations for ground truth data is typically a highly labor‑intensive and challenging process that accounts for the majority of the time spent. As one MAI-PhD explains, “[b]efore we start developing, it takes months to prepare the data. Training a model takes like a week, but just preparing the entire data[set] takes a lot of time: 80% of the time just goes into cleaning the data” (002). Contemporary deep-learning techniques require models to be trained and evaluated on large amounts of annotated medical data.

Medical data interpretation requires specialized expertise, but limited time, resources, and expert availability constrain such large-scale annotation. Three MAI-PhDs, whose work involved creating segmentation masks of intravascular structures and lesions in high‑resolution intracoronary OCT images from a specialized cardiac catheterization lab, were concerned about this lack of expertise. One MAI-PhD claimed that “[t]he problem with OCT data is that there are not enough people who have the knowledge that can annotate this type of data in the first place” (009, translated by the first author). The three were therefore required to conduct the annotations themselves, producing segmentation masks for many scans “from scratch” (007, translated by the first author), which took up a lot of time: “[w]hen I started it took me 1 h to annotate one frame. By now I think it takes me about half an hour. That is the reason why we only annotated 2% of the data.” (009, translated by the first author).

For these reasons, and similar to other ground truth studies (Jaton 2023; Catanzariti 2025; Högberg 2025), different kinds of uncertainty and dissonance are intrinsic to the production of data labels, and many participants resisted the promise that one can strive for “an objective ground truth” (009, translated by the first author). As one MAI-PhD working on segmentation masks for bone structures and knee implants in CTs argued: “[y]ou can just never be sure if it’s correct […]—there’s nothing like a real ground truth” (003). However, in constructing the required data labels that are necessary for training and evaluating models, they must make a call on what they “think is the correct answer” (004), eventually settle on uncertainties, and make sometimes arbitrary decisions. We argue that it is in these moments of temporal stabilization that annotating practitioners create the epistemic spaces we call *zones of (un)certainty* in medical ground‑truth production.

In what follows, we unfold three interconnected *zones of (un)certainty* through which epistemic certainties, uncertainties, and dissonances in medical data annotation are operationalized to stabilize ground truths for medical AI. The first zone is an epistemic space in which ground-truthing, and the stabilization of uncertainty are approached spatially. Practitioners treat data points as layered entities and move back and forth through the data, decomposing and recomposing layers of knowledge. By adding further spatial dimensions, such as previous cases, alternative views, or metadata, they construct a more comprehensive decision environment and work toward “good enough” certainty. The second zone is a time–space in which uncertain and divergent labeling decisions drift through human and non-human temporal practices. The annotators’ micro-judgments are shaped by ongoing negotiations among themselves, colleagues, medical experts, AI suggestions, and automated evaluations. Within these “zones of drift”, temporarily stabilized decisions remain fragile and open to reconfiguration as circumstances, interactions, technologies, and routines shift. The third zone is an epistemic space in which ambiguous labels and indeterminate cases are worked into singular categories through what we call thresholding practices. Here, zone decisions are not made at a distinct boundary but transversal and across a spectrum, where competing possibilities are gradually compressed into a “certain enough” classification.

These zones of (un)certainty overlap yet remain distinct, each revealing different socio-technical knowledge spaces through which medical ground-truth data is constructed and stabilized. Together, they show that developing ground truth for medical AI is not only a matter of certainty or uncertainty, but of actively creating zones where annotators negotiate (un)certainty to reach required outcomes. By examining these zones in detail, we can better understand how data annotators transform, stabilize, and materialize epistemic uncertainties and dissonances through sets of routine and pragmatic practices to settle on ground-truth decisions. This, in turn, illuminates the socio-technical logistics through which uncertain and divergent micro-judgments are routed into actionable ground truths for medical AI.

### Decomposing and recomposing layers of knowledge

Grounded in a spatial approach to the data they were annotating, participants would compose and recompose layers of knowledge about the data and establish an epistemic zone in which they would become increasingly certain and reach a decision to apply a specific ground-truth label. Following Aradau and Blanke’s (2022, p. 16) analysis of algorithmic knowledge practices, AI annotators “simultaneously split up [data] components” and “recompose them” into an “integrated, but dispersed workflow” established by individual practices. The creation of a layered zone for knowledge production by re- and decomposition—step-by-step ruling out small uncertainties to reach a stage in which annotators become somewhat “certain enough”—became immediately apparent as both annotating MD and MAI-PhDs explained making use of the software features and tricks to analyze the visual data thoroughly. Reporting on a project of annotating 12-lead ECG signals for the development of a machine learning application that detects and predicts atrial fibrillation (irregular heart rate) with ICU Patients, one MD, e.g., recounts: “I would work with two screens. On the left screen, […] I could see the ECG and on the right screen I would have background medical information on medical ECG analysis in case I would be uncertain about something” (005, translated by the first author).^2^ Specifically focusing on the uncertain cases, the annotator would start by “zooming into the system” (005, translated by the first author) and take a screenshot to get a more detailed view of the ECG signals. Because the rhythm (e.g., direction and consistency) and the thickness of the lines representing the ECG signals are crucial for classifying a data point as atrial fibrillation or not, a close-up view enabled a more nuanced analysis. It created an epistemic space in which the annotator could gradually become more confident in their judgment—especially since the most important indicator of atrial fibrillation is irregular signal motion in the image.

This layered decomposition and recomposition of knowledge structures about the data to reach a zone where annotators would be confident enough to make a final decision on a data label became particularly evident in conversations with MAI-PhDs annotating medical images for their own research projects. As imaging data such as CT, CTA, or OCT are multi-dimensional entities, MAI-PhDs conducting annotations for these data types would apply different views to construct a more comprehensive “image of the data” from which they could extract the necessary forms of knowledge to inform their judgment. By doing so, these annotators do not just analyze the surface artifacts but instead “archeologically” dig deep into the data, penetrating downwards through the various imaging layers and accessing their spatial repositories. Working on a project to annotate artery bifurcation points^3^ in Run-off CTAs,^4^ one MAI-PhD explained:

“I always struggle making the decision if some patients get a bypass that is anatomic, which means that it is roughly in the same position as the original artery through which the blood flow is completely obstructed. I then struggle to characterize this as the artery or not, because it looks similar enough, but it is in a slightly different place.” (004)

In response to this type of uncertainty, the annotator would “play around with the views” (004) of the images. This means that they would shift perspectives between the Axial and the Coronal views to obtain a more detailed account of the arteries’ positions in the images and to gradually “connect all of the pixels that have similar brightness” (004). This practice allowed them to construct a spatial view which informed their final decision. In this case, the annotator decided that arteries whose positions did not appear to have shifted, but whose pixel brightness differed too much, should be treated as the original vessels. Arteries that did appear to have shifted position were instead interpreted as new branches that had bifurcated from the originals in the image.

Adopting a different strategy, medical data annotators also generated zones of stepwise certainty by enriching the data with additional spatial dimensions, drawing on previously annotated datasets, metadata, and documentation during label production. To this end, annotators look beyond the data they are currently labeling and excavate it archeologically, both horizontally—e.g., by drawing on previously annotated datasets with comparable patient data—and vertically, by consulting metadata and documentation. As one annotator working on calcium segmentation in coronary CTs put it,

“I look at annotations which were done on a previous dataset done by multiple experts, and [...] see if it matches with my case. And then I can make a fair judgment. It is like finding the same example where the calcium is in the same location, so it does not always work, but for some classes, those very rare classes, I am more likely to find okay: ‘this is what it looks like, and this is what I annotated’.” (002)

The same annotator explained keeping an Excel sheet where they would add comments to keep track of their ground-truth decision-making process. Such comments involved information about whether “[the] scan should be reviewed by an expert,” (004), if the annotator was “unsure about some areas” (004) or if something was “modified [in] the annotations” (004). Similarly, others described constructing a “holy Excel”-document (009) containing a list of moments of uncertainty and doubt in drawing segmentation masks for OCT images (008; 009) or developing a “Standard Operating Procedure (SOP))" (006; 010). This document would keep track of all collective “work agreements” (010) regarding annotation decisions that could be consulted by all project participants in case of uncertainty and discussion. The SOPs, shared spreadsheets, inter‑expert consultations, and AI‑based checks can be seen as small‑scale instances of Cambrosio et al.’s (2006) regulatory objectivity in the making. They are conventions about what will count as a correct label that are collectively produced and iteratively adjusted.

Taken together, annotators of medical data thus construct dynamic (un)certainty zones in which they epistemically transform emerging uncertainties and dissonances arising from data interpretation into temporally stabilized data labels. These zones are built step-by-step by expanding the data’s spatial dimensions and integrating previous datasets, metadata, and documentation into a unified, multi-layered annotation. In doing so, the zone becomes a logistical tool that traces exactly how both human and AI reasoning navigated ambivalence to reach a definitive ground truth that can be used for further model development.

### Drifting epistemic decisions

Considering the temporal aspect of annotation work and the production of ground truth data, an additional zone of (un)certainty emerged as annotators would create decision-making zones in which their individual micro-judgments would “drift” over time and temporarily materialize a “certain” decision in the form of a single label.^5^ These epistemic zones would develop through repetitive practice including continuous individual annotator negotiation—e.g., by “[always] go[ing] past the same annotation twice to make sure [not to] miss or mistakenly annotate something [that] shouldn’t be there” (011)—as well as through negotiations with other human actors like colleagues and medical experts and also with non-human actors (e.g., AI systems’ suggestions and evaluations).

One annotator drawing segmentation masks in CTs, e.g., explained evaluating their work to become more certain about it:

“At one point, I realised: ‘oh, maybe I do not have a full understanding of [what] an implant actually looks like in real life.’ So this time I talked with clinicians and showed them screenshots and was like: ‘hey, this is what I would segment, would you agree with that?’ and then they would be like ‘no, that is not part of the implant’ or something like that. So [...] with that, over time I kind of developed an annotation protocol for myself” (003).

The MD-PhD working on classifying ECGs according to patterns of atrial fibrillation described a similar process. Yet this time, the aim was to become confident that the data were not to be used for further training:

“A few times, I also asked colleagues: ‘Can you please have a look at this ECG, what do you think?’ If all three had different opinions, I just entered that we could not judge the data and make a decision [on the label]. Then the data [point] is also useless.” (005, translated by the first author).

The decision not to label the data at all and classify it as useless was reached through the establishment of an epistemic zone of drift in which the assemblage of annotators’ individual judgments and the various steps of consulting and considering colleagues’ opinions resulted in the construction of a confident judgment about the data.

The emergence of this type of temporal decision-making zones also became prominent through conversations in which participants explained using AI systems to evaluate their annotation judgments. For example, in the project where annotators drew segmentation masks of intravascular structures and lesions in cardiac OCT scans, one participant had produced the initial segmentation masks for 600 OCTs “from scratch” (007, translated by the first author). A colleague explained that “[a]fter that, we trained the model to evaluate the predictions based on those manual annotations” (007, translated by the first author). In doing so, the group reached a point where the model performed relatively well, so they “only need[ed] small corrections in [their] annotations” (007, translated by the first author).

Taking this practice one step further, the MD-PhDs annotating electronic health records for a large national ICU database (006; 010; 012) described building an LLM that provides annotation suggestions to map 73,334 hospital variables to a database structure based on a series of standardized ICU medical concepts. Beltrame et al. (2026) characterize contemporary biomedical platforms as organized around “nested multifocality”, in which diverse gazes, from computational to clinical, operate across multiple, hierarchically related focal layers. Our interviews have provided us with evidence of how the LLM gaze is now part of this:

“These suggestions are based on a language model that predicts the best connection between our protocol of standardized medical concepts and the hospital’s data variables. This process is being monitored by a minimum of two PhDs. The first maps the variable toward a standardized concept in our database and the second validates that judgment or not. If there is a discrepancy between the two PhDs, they will have a discussion first, and if they do not come to a final decision we reach out to an intensivist located at the hospital of the data’s origin” (006, translated by the first author).

As such, the group is moving through a zone in which their opinions are temporally reshaped by the LLM’s suggestions and by the conversations between the annotating PhDs and the ICU doctor. This constitutes a particular configuration of nested multifocality in Beltrame et al.’s (2026) sense, where the clinical, algorithmic, and annotator perspectives are continuously realigned in real time, as LLM outputs redirect attention and make interpretations more or less salient. The gaze of the LLM is first assessed by two PhD annotators and, if necessary, escalated to an intensivist within a layered clinical and expert framework. Simultaneously, the use of the LLM within this zone significantly speeds up the process. One group member explained: “We will soon receive a batch of 60,000 data samples which we can map within about three weeks. […] Imagine we would do this manually using an Excel sheet, it would be 100 × slower.” (010). As combinations of LLM suggestions, human micro-judgments, and PhD-expert cross-validations, these zones of drift can lead to the space where a “certain enough” consensus can be stabilized. The final label is decided on relatively quickly while staying within a project’s time constraints.

However, the temporal circumstances that allow these zones for stabilized ground‑truth decisions to unfold also make them inherently hard to maintain. This became evident when one MD-PhD who annotates blood pressure data for an AI system predicting postoperative ICU complications noted how difficult it was to capture the transient nuances of the process: “[T]he truth is never absolute, […] maybe you are not even looking at the data in the same way, […] this could for example be because someone forgot to wear their glasses” (001). Others mentioned that decisions could quickly drift into different directions, because annotators “are just in the zone” and “do not realize that you are actually annotating this class with a different label then you were supposed to” (002). When they look at the data again two days later, they may “come to a different decision” (007, translated by the first author).

Through current human and non-human practices, annotators navigate a temporal zone of drift to build consensus and produce ground truths. Their micro-judgments are steered by ongoing negotiations with themselves, colleagues, medical experts, AI suggestions, and evaluations. Within this interval, final labels emerge not only from individual data entities, but from the continuous interplay between annotators, experts, and AI systems—especially via interval evaluations and LLM-based label suggestions, as in the ICU database annotation project. Thus, annotation moves through a temporal zone where human–machine judgment is dynamically routed, drifting decisions in specific directions. For a given validity interval, labels become products of temporal socio-technical judgments shaped by AI research, clinical interests, practices of scientific consensus, and AI influence (e.g., LLM suggestions or AI-based validation). This zone is a “melting pot” that can only be temporarily stabilized through these socio-technical assemblages, allowing for negotiations between AI research and medical practices until a certainty threshold is met.

### Epistemic thresholds

When examining boundary-setting practices in decisions about applying specific data labels, the final zone of (un)certainty became evident. Rather than drawing sharp, line-like boundaries, annotators created epistemic zones that functioned as thresholds along a spectrum, where multiple judgments, possibilities, and negotiations gradually transformed into a single ground-truth labeling decision. Following Gabrys and Yusoff (2012, p. 6), we refer to these types of zones as epistemic thresholds. These offer annotating participants a way to bring “together sites of encounter, transformations [and] uncertainties.” Contingent on the specific parts of the process that annotators were working on, we observe two strategies for threshold-setting. The first one implies the creation of epistemic thresholds that transform data-specific (spatial–temporal) judgments to stabilize individual or collections of ground-truth decisions. The second produces a zone in which participants set thresholds for validating the quality of annotated data.

The production of data-specific annotation thresholds as “zones of transition” (Aradau and Blanke 2022, p. 193) became particularly apparent when annotators described reaching decisions using medical decision-making scales, gradients derived from specialized medical protocols, or (again) by digging deep into the data. For example, the participant working on annotating blood pressure data to predict complications in postoperative ICU patients discussed the so-called “complication registration” scale (001, translated by the first author). This scale influences the uncertainties in decision-making, whether the type of complication will be annotated in the first place: “[t]here are different grades, but we are only interested in everything above grade 3a for the model that we are developing” (001, translated by the first author). In determining this threshold, the annotator explained they would look at the severity of the complication:

“Grade 1 refers to the first level of deviation from the norm […]. It could be that someone becomes very nauseous directly after surgery. […] Imagine someone developed atrial fibrillation or a small, infected wound. This does not necessarily have to be something serious, but it deviates enough from the medical norms of how the patient is supposed to feel. We would call this grade 2. Grade 3 means a [medical] intervention was necessary. […] Grade 4a and 4b mean that [the patient] has been admitted to the ICU because of multiple dysfunctional organs. Grade 5 means [the patient] passed away. Grades 1 and 2 are very common, but [these complications] are less important when one aims to predict the likelihood of a severe complication. Grade 3 provides us with more information, and therefore, we decided to do it in this way” (001, translated by the first author).

This discussion about labeling complication types—setting thresholds for which complications qualify for inclusion as dataset annotations—offers insights into how this epistemic zone is operationalized as annotators articulate its internal and external boundaries.

Working with very different data modalities, such as production of in/outside boundaries to reach a level of certainty, also emerged in conversations with other annotators. For example, the participant annotating calcium segmentation masks in coronary arteries described how they would determine the trustworthiness of the data by going through all the layers of entire CT scans looking for artifacts: “[I would] go through [an] entire CT scan,” (002) feeling “like [a] few slices were missing” (002) because there seemed to be a lot of movement between the slices and that did “not make sense” (002). The data was acquired using “a very old scanner where the patient was put in place while doing the scan, one slice was taken, and then the patient was moved instead of the scanner moving” (002), causing uncertainties in the scans that would make them less suitable for drawing reliable segmentation masks. In contrast, the same annotator explained coming to more certain annotations when the scan would have been acquired, while “the patient [was] holding breath” (002), because “the [breathing] artifact was reduced” (002). This archeological approach of going through scan layers thus lets the annotator produce boundaries according to thresholds, transforming their judgments across the different layers into threshold decisions that determine the scale of certainty for different annotations.

At the microdata level, the team of MAI-PhDs working on annotating segmentation masks of intravascular structures and lesions in cardiac OCT scans would describe looking for highly specific light–dark transitions in the data to increase confidence. They could then set a boundary to stabilize a decision and draw a segmentation mask: “[e]specially [with the annotation of] lipide, […] you look for the moment of the largest transition—the largest transition from light to dark” (007, translated by the first author). As another team member notes, these threshold judgments were reinforced in team discussions to improve certainty about the segmentation mask in question: “[w]ith all the edge-cases, we would always have discussions, also with doctors” (008, translated by the first author). The team negotiations then led the annotator to transform such a collection of opinions and to use the feedback to create a zone in which they became increasingly confident in their ground-truth decisions over time. As the same team member explained, “even though I am still uncertain about some cases, overall, I have become more certain [about them] […]. One can only remain uncertain about the transition on a pixel-level, where to specifically draw [the segmentation mask]. But that is less important than missing an entire [intravascular] structure” (008, translated by the first author).

The production of zones in which annotators would set thresholds to validate the quality of entire labeled datasets would become clear in two ways: first, annotators (002; 003) would describe setting temporary threshold boundaries that would determine whether the amount or level of error in the annotations actually mattered to the quality of the ground truth. As one of them argued:

“If I see in my annotations there are like 20 scans which had an incorrect label, that means the annotation quality is really bad, but if out of 600 scans one or two scans have the wrong label, that is fair it might be due to the repetitive nature of the task […]. There is a certain threshold of quality within the annotated data that can make the model account for one or two errors. Like, if it’s very small, I do not think it matters to put that much additional effort into doing something to make it more precise.” (002).

These zones as thresholds for determining the quality of annotated data would thus be drawn according to the impact potential errors would have on model performance during evaluation.

Second, over the course of annotating the data, others would additionally perform several rounds of evaluations to become certain enough about their ground truth decisions:

“In the world of coronary OCT data, there remain a lot of uncertainties. However, when I look at how often our model performs well on the [annotated] data, this means that we actually annotated [that data] correctly. I reckon this provides some real potential and I think this also averages out the errors and the uncertainties [that remain part of the process]” (007, translated by the first author).

As such, the annotators would use computational efficiency to immediately test their model on annotated data and draw the in/ outside boundary that separates the zones with “certain enough” annotations from the rest.

Whether through the transformation of data-specific spatio-temporal judgments or the validation of data quality, annotators navigate epistemic zones as thresholds for setting decision-making boundaries. Within these specific zones, annotators operate at both micro- and macro-data levels. On the one hand, they combine their archeological data and practices to “dig deep” with collective negotiations to set thresholds. On the other hand, annotators reach threshold decisions by determining the potential impact of annotation errors on model performance and by operating intermediate evaluations to improve annotation quality. As such, this zone of (un)certainty represents the knowledge space in which annotators move between the micro and the macro, and between human-computational judgments, to reach a certainty threshold and materialize ground-truth decisions.

## Concluding discussion

Extending critical data, AI, and STS research on the role of uncertainty in producing ground-truth data (e.g., Cambrosio et al. 2006; Jaton 2023; Catanzariti 2025; Högberg 2025; Beltrame et al. 2026), this article introduces the concept of *zones of (un)certainty*. With this framework, we empirically capture the epistemic time-spaces through which medical data annotators work and transform uncertainty and dissonance to stabilize and materialize ground truths for medical AI. This framework is important because, unlike in broader AI sub-fields such as computer vision, medical data annotation depends on domain experts whose labor is costly, time-intensive, and embedded in complex clinical knowledge. The process of producing ground-truth labels is shaped by epistemic uncertainties, situated judgments, and incommensurable ways of knowing medical phenomena, reflecting the multiplicity of medical bodies across practices and contexts (Mol 2002). Consequently, annotation work is inherently unstable and negotiated, making the construction of reliable and actionable ground-truth datasets a persistent challenge with important implications for bias and model performance in medical AI (Luitse et al. 2025; Högberg 2025).

Despite ongoing challenges related to cost, ambiguity, and epistemic uncertainty in medical annotation practices, medical AI systems continue to rely heavily on manually constructed ground-truth datasets. To develop model training and evaluation, annotations must be finalized, and contingent medical uncertainties must be transformed into sufficiently stable and actionable decisions to produce the final set of reference labels. To trace how annotators carry out this work, our framework allows us to unfold the temporal and socio-technical zones in which epistemic uncertainties and dissonances are negotiated, organized, and temporarily and spatially stabilized.

*Zones of (un)certainty* illuminate how mundane scientific practices and situated ways of knowing across different disciplines (e.g., AI and medicine) are aligned and standardized to produce large quantities of medical ground-truth data critical to AI systems. These zones are not merely sites of data labeling, but socio-technical epistemic time-spaces in which certainty, uncertainty, and dissonance are negotiated and stabilized (Cambrosio et al. 2006), and where ground-truth data pass diverse thresholds to be considered “certain enough” for model training and evaluation. To develop this concept, we have built on Galison’s (1997) concept of trading zones, which highlights coordination among members across different scientific disciplines to develop shared understandings. We have combined Galison’s work with Andrew Barry’s (2006) technological zones, which foreground alignment through infrastructures, standards, and protocols. *Zones of (un)certainty* subsequently extend both ideas and allows us to empirically focus on temporal-epistemic spaces where medical data annotators operationalize the mundane and where their improvisational knowledge practices transform uncertainties to settle on ground-truth labels. By focusing on this aspect of ground-truth data production, this perspective offers a novel lens for understanding how certainty, uncertainty, and dissonance are continuously enacted, negotiated, temporally stabilized, and made actionable in domains such as medical AI. Thereby, we illuminate the socio-technical logistics through which uncertain and divergent micro-judgments are routed into workable ground truths. This is empirically substantiated across our three empirical-analytical sections.

In our empirical account of annotator micro-judgments and ground truth decision-making practices from AI projects in two Dutch medical centers, we identify three interconnected zones of (un)certainty: first, we have shown that ground-truth construction unfolds within epistemic spaces where uncertainty is approached through spatial practices of engaging with the data. Annotators treat data not as isolated points but as layered, relational entities, moving archeologically back and forth across different dimensions to decompose and recompose relevant information. As such, they operate within a layered zone of knowledge production, progressively ruling out minor uncertainties until they reach a point at which decisions become “certain enough”. This became particularly clear as annotators of imaging data would dig deep into the data layers to extract knowledge, moving across slices to construct a comprehensive view and settle on ground-truth decisions. In addition, they incorporate additional spatial perspectives—such as prior cases, alternative views, or contextual metadata—to construct richer decision environments for ground-truth labels.

Second, our analysis highlights temporal zones in which human–machine annotation decisions are constantly (re)routed and shaped, drifting them into particular directions. Within these zones, micro-judgments continuously evolve through ongoing negotiations between annotators themselves, between annotators and medical experts, and engage with AI-based suggestions and evaluations. The use of LLM-generated label proposals in ICU annotation workflows for ground-truth construction requires particular attention when further researching such temporal decision-making dynamics as they introduce continuously updating, non-human inputs into the annotation process, reconfiguring how future medical uncertainties and interpretations are interpreted, steering decisions through their predictive suggestions. These zone-based interactions thus introduce temporal dynamics in which decisions are repeatedly revisited, adjusted, and recalibrated through iterative validation practices, interval evaluations, and feedback loops. This allows annotators to steer, test, and refine their judgments over time. As a result, even stabilized labels remain provisional and open to reconfiguration, as human–machine judgments continuously shape how decisions are routed and settled. In this sense, annotation unfolds within the temporal “zones of drift” in which socio-technical judgments are dynamically assembled, and where ground-truth labels stabilize and emerge as the outcome of ongoing negotiation between AI research objectives, medical reasoning, and the increased influence of AI systems within the process.

Lastly, we have identified a third type of epistemic zones in which uncertainty and dissonance are stabilized through thresholding, which allows for the gradual transformations of judgments and negotiations into ground-truth labeling decisions or for validating the quality of annotated data. Within these threshold zones, we showed that annotators do not set fixed boundaries but operate at a micro-level to reach decisions by using medical decision-making scales, gradients derived from specialized medical protocols, or through more archeological data digging. This was specifically highlighted by both MD-PhDs in the ICU and MAI PhDs in Cardiology, which described operating a “complication registration scale” or looking for the most prominent light–dark transitions in the imaging data on a pixel-level to become more certain about their judgments and include the data in their ground-truths.

In conclusion, our conceptualization of zones of (un)certainty in ground-truth construction not only allows us to account for how ground truths are stabilized but also opens up a broader analytical vocabulary for studying how uncertainty and dissonance are organized in complex socio-technical systems such as AI pipelines more broadly. In medical AI and related development and application domains, zones can be understood as evolving epistemic infrastructures whose spatial dimensions and boundary-setting thresholds are reconfigured over time as data, AI and domain expertise, and computational infrastructures are mobilized and integrated. This invites further inquiry into how such zones are designed, governed, and contested, not just for ground-truth production but also for other practices of knowledge production in AI development. Attending zones of (un)certainty more broadly would provide a way to illuminate how epistemic authority is differentially distributed between human and non-human actors, and how these configurations shape what comes to count as reliable and actionable knowledge across the AI lifecycle. In this sense, zones of (un)certainty reposition not only ground truth but AI knowledge production more generally as a temporally situated and contested achievement, opening critical questions about how knowledge, authority, and uncertainty are organized for contemporary AI-based medicine and beyond.

## Data Availability

The data generated and analyzed during the current study are not publicly available because they contain information that could compromise the privacy of research participants. In accordance with the European General Data Protection Regulation (GDPR) and the conditions under which participants provided informed consent, the data cannot be shared.

## Footnotes

1 See, e.g., Pratt (1991), Galison (1997), Barry (2006), Collins et al. (2007) for prominent conceptualisations of zones in STS, as well as Star and Griesemer (1989) for related work.

2 “12-lead” means that the ECG consists of an image of 12 signals that together represent the electrical activity within different parts of the heart.

3 Artery bifurcation points are points at which a single, main artery divides or forks into two separate branches.

4 This imaging technique allows for the visualization of the aorta and lower limb arteries. The annotation task is conducted as part of a project on the detection of characteristics of primarily Peripheral Artery Disease (PAD), characterized by “the accumulation of calcified and non-calcified plaque in the arteries of the legs” (004).

5 In medicine, annotations are always temporal. An annotation that is considered static may be particularly undesirable, as data may also follow a patient treatment trajectory. A tumor labeled on January 21st, for example, has a strict validity interval. By February 21st, after chemotherapy, that annotation might be obsolete and therefore less valid in particular situations.
